# Technical and Clinical Outcomes at a Thrombectomy-Capable Stroke Center in Poland in the Context of the Center’s Growing Experience, Expanding Treatment Guidelines and the Rise in Acute Ischemic Stroke Patient Volume: A Comparative Analysis of Initial and Subsequent Endovascular Procedures

**DOI:** 10.3390/life16020304

**Published:** 2026-02-10

**Authors:** Artur Dziadkiewicz, Krzysztof Pawłowski, Anna Podlasek, Michał Sulkowski, Krzysztof Gawrych, Marek Szołkiewicz

**Affiliations:** 1 Department of Neurology and Stroke, Florian Ceynowa Hospital, Pomeranian Hospitals, 84-200 Wejherowo, Poland; 2 Department of Cardiology and Interventional Angiology, Kashubian Center for Heart and Vascular Diseases, Florian Ceynowa Hospital, Pomeranian Hospitals, 84-200 Wejherowo, Poland; krzysztof.pawlowski@wp.pl (K.P.);; 3 Image Guided Therapy Research Facility, University of Dundee, Dundee DD1 4HN, UK; 4 Department of Cardiology, Specialistic Hospital, 82-300 Elblag, Poland

**Keywords:** thrombectomy, ischemic stroke, learning curve, endovascular reperfusion, thrombolysis, stroke treatment time window, large vessel occlusion

## Abstract

(1) Introduction. To improve access times and provide effective treatment to the growing patient population with acute stroke due to large vessel occlusion (LVO), thrombectomy-capable stroke centers (TCSCs) should be made an integral part of hospital infrastructure in Poland. The geographical proximity of thrombectomy-capable centers and recently extended treatment time windows will considerably increase patient numbers, decrease patient disability, and reduce the costs of long-term care. (2) Aim of the study. This study investigates the clinical outcomes, time metrics, and angiographic data of a cohort containing 250 thrombectomy patients at a single TCSC in Poland. We measured performance against data from the national database during two crucial time intervals: at the very beginning of the center’s service and after the involvement of a new operator. This study considers concurrent modifications in qualification guidelines, the TCSC’s transition from a ‘direct-admission-only’ to a ‘drip-and-ship’ model, and the learning curve of the interventional stroke team. (3) Methods. A retrospective analysis was conducted on 250 patients treated from August 2020 to May 2025 at a newly established TCSC. The cohort was dived into 2 subgroups: an initial group of 100 patients, whose treatment corresponded to the involvement of a new, previously trained on-site operator and the establishment of 24/7 service, and a group of 150 patients who received later treatment. Additional comparisons were made between a cohort of directly admitted patients and those treated under the drip-and-ship model. The results compared between patients treated with early and expanded time windows. (4) Results. Significant differences were observed between the first 100 and subsequent 150 patients in terms of admission scheme (97% vs. 70%, *p* < 0.0001), extended time window treatment (8% vs. 17.3%, *p* < 0.05), and intravenous thrombolysis treatment (81% vs. 65.3%, *p* < 0.01). Improvements in time intervals and procedural factors were noted in the second group, reflecting the operator’s increased experience (groin-to-first pass time: 27 vs. 23 min, *p* < 0.05). A comparative analysis between the direct admission and drip-and-ship models revealed extended time intervals in the latter (door-to-groin: 110 vs. 159 min, *p* < 0.001; door-to-recanalization: 158 vs. 200 min, *p* < 0.001; door-to-CT: 9 vs. 16.5 min, *p* < 0.001; and door-to-IVT: 21 vs. 43 min, *p* < 0.001). Patients in the extended time window exhibited lower intravenous thrombolysis rates (78.2% vs. 29.4%, *p* < 0.0001) and prolonged door-to-groin (117.5 vs. 150 min, *p* < 0.005), door-to-CT (10 vs. 19.5 min, *p* < 0.01), and door-to-IVT (25 vs. 77.5 min, *p* < 0.001) times. No significant differences were found in complication rates, clinical outcomes, or mortality between the analyzed subgroups. (5) Conclusions. The present data demonstrate favorable clinical and angiographic results among acute LVO stroke patients at the newly established TCSC, both at the onset of the mechanical thrombectomy service and after the involvement of a newly trained operator. Even when treating patients with prolonged times due to transportation and late window qualification, we observed favorable clinical outcomes and low rates of complications. The results achieved in our TCSC compared with the national data suggest that TCSCs could potentially play an important role within the overall endovascular treatment system for acute ischemic stroke patients in Poland.

## 1. Introduction

Mechanical thrombectomy (MT) alone or in combination with intravenous thrombolysis (IVT) is a recognized class I A treatment for acute ischemic stroke (AIS) caused by large vessel occlusion (LVO) [1]. Thrombectomy-capable stroke centers (TCSC), established as lower-volume support centers within the general stroke system, may play a significant role in endovascular stroke treatment in Poland [2]. Due to clear inadequacies in the interventional stroke treatment system in Poland, where a pilot program was active for many years, a number of neurology and stroke departments in collaboration with experienced interventional operators (e.g., cardiologists, neurosurgeons, vascular surgeons, or radiologists) have established low-volume thrombectomy centers providing mechanical thrombectomy to patients in their regions [3,4]. The Wejherowo Hospital Department of Neurology and Stroke, in cooperation with the Department of Cardiology and Interventional Angiology, launched a thrombectomy program dedicated to treating AIS patients from the Northern Kashubia region who were admitted to the Emergency Unit at our institution. The conversion of a primary stroke center into a thrombectomy-capable center was fundamentally linked to the duration of the center’s operation and the staff’s experience. This process was influenced by the unavoidable learning curve of all medical and non-medical personnel involved in the entire procedure, namely radiologists, operators, nurses, radiography technicians, and supporting personnel [5]. Additionally, the center’s experience and procedural aspects of endovascular stroke therapy were significantly influenced by the expansion of the patient qualification guidelines, which included European and national official guidelines, as well as evidence-based medical data derived from randomized clinical trials and other sources of professional knowledge. The modifications to reimbursement for endovascular stroke therapy in Poland necessitated the integration of thrombectomy centers with other primary stroke centers through a drip-and-ship mechanism, thereby improving cooperation and increasing patient volumes in our TCSC. However, this change also resulted in certain drawbacks, such as extended time intervals for treatment. The recently published Polish national report on stroke treatment, issued by the Narodowy Fundusz Zdrowia (NFZ, National Health Service), offers an opportunity to compare outcomes from the TCSC in Wejherowo with those from the Pomerania region and the entire country.

The primary objective of this study is to examine observable changes in the practice and development of a newly established thrombectomy-capable stroke center (TCSC) in Poland, particularly in relation to changes in the mechanical thrombectomy qualification guidelines, such as the extension of the time window and the acceptance of patients with lower ASPECTS scores. Additionally, this study explores the clinical and technical aspects of the TCSC’s transition from a ‘direct-admission-only’ model to a ‘drip-and-ship’ model, as well as the mechanical thrombectomy learning curve and acquisition of experience by the interventional stroke team. The present analysis is based on a comparison between two cohorts of patients, the first consisting of one hundred patients treated by only one certified operator at a newly established thrombectomy center and the second comprising one hundred and fifty patients treated at the same TCSC in Wejherowo by a team of two operators, one of whom had recently obtained the necessary experience in relevant procedures. The analyzed parameters include the baseline characteristics of both groups, time intervals, complication rates, and final outcomes. In this way, we compare the two groups and seek to uncover potential differences in outcomes due to the existing learning curve. An additional comparison was made between the aforementioned results and data from the national stroke database of the Polish Health Fund (NFZ). The study’s secondary aim is to examine the function of TSCS within the Polish stroke care system, focusing on how extending the time window, patient qualification processes, and transfer delays affect the ultimate clinical outcomes. Describing the learning curve, particularly in TSCS involving a cardiology team, could significantly impact the design of neurointerventionalist training programs.

## 2. Materials and Methods

The study is a retrospective analysis of the total population of 250 patients treated at the Thrombectomy-Capable Center in Wejherowo (Department of Neurology and Stroke, Neurointerventional AngioSuite in Department of Cardiology and Interventional Angiology, Kashubian Center for Heart and Vascular Disease) from August 2020 to May 2025. The total population of patients was divided into two subgroups: The first contained 100 patients treated endovascularly between August 2020 and May 2023, and the second contained 150 patients treated from May 2023 to May 2025. The rationale for subdividing and analyzing these subgroups was based on an observable substantial increase in the number of treated patients due to the introduction of 24/7 service, along with the implementation of guidelines for extended time windows and the establishment of collaborative efforts with other primary stroke centers (PSCs). Patients presenting with stroke symptoms and LVO (defined as occlusion of the internal carotid artery; M1 or proximal M2 occlusion of the middle cerebral artery; occlusion of the basilar artery; and, in select cases, occlusion of A1 or A2 of the anterior cerebral artery, vertebral artery, and P1 of the posterior artery) were subjected to endovascular treatment, with or without intravenous thrombolysis (IVT), following an assessment by a stroke or interventional neurologist, radiologist, and interventional cardiologist. Prior to the intervention, all patients underwent plain computed tomography (CT) imaging and computed tomography angiography (CTA). MRI scans and computed tomography perfusion (CTP) were conducted among a select group of patients in cases of extended time window protocols (see Table 1). The early time window and primary MT inclusion criteria were as follows: over 18 years of age, a National Institutes of Health Stroke Scale (NIHSS) score of at least 6 or isolated aphasia, and prior functional independence with a modified Rankin scale of mRS 0–2. Additional criteria for extended time windows (based on the criteria of the Wake-up, Dawn, and Defuse clinical trials) are presented in Table 1 [6,7,8]. Before the procedure, all eligible patients received intravenous thrombolysis with one of two thrombolytic agents: alteplase or Tenecteplase (with a weight-adjusted dose). Endovascular procedures were performed by either the interventional cardiologist (experienced in neurointerventional procedures) or the interventional neurologist. The first one hundred cases were performed by a cardiology-based independent operator at our newly established thrombectomy center, while the following 150 cases were performed by a mixed team consisting of either the aforementioned interventional cardiologist or an interventional neurologist who recently became an independent operator. The default method of thrombectomy was SAVE (a combination of an aspiration catheter and stent retriever). However, at the discretion of the operator, ADAPT (aspiration only) or stent-retriever-only (SR) techniques were utilized. A balloon-guiding catheter (BGC—FlowGate2^®^ or Merci^®^, Styker, Fremont, CA, USA) or vascular sheath (NeuronMax^®^, Penumbra, Alameda, CA, USA) was also used at the discretion of the operator, depending on the age, aortic arch type, and expected problems with set stability.

All time intervals were collected before, during, and after the procedure. The initial time point, named as “door time”, was defined as the patient’s admission time to the first stroke center, either primary (without a thrombectomy service) or secondary (direct admission to the TCSC). The next recorded time points were as follows: CT (time of neuroimaging),LVO (time of large vessel occlusion identification and pre-qualification to EVT), groin (time of groin/other access site puncture); first pass (time of crossing the clot with microcatheter/stent retriever or distal access catheter); recanalization (time of the last pass or time when final forward flow in the affected vessel was achieved). The clinical neurological state was assessed by a stroke neurologist using the National Institute of Health Stroke Scale (NIHSS) at set time points: before the procedure, 24 h after MT, and on discharge. The baseline modified Rankin Score (mRS) was assessed by the neurologist based on the patient’s history, while the next measurement of the disability score was performed on discharge and at a three-month follow-up. Mortality was assessed at the three-month follow-up. After the procedure, the radiological outcome was measured visually by two different operators with a Modified Thrombolysis in Cerebral Infarction (mTICI) scale. Here, mTICI 2b-3 was identified as satisfying the criteria, while mTICI 3 was considered an excellent radiological outcome. Hemorrhaging of the brain parenchyma was classified based on ECASS (European Cooperative Acute Stroke Study) [9], with PH2 considered the most severe and consequential. Symptomatic intracranial hemorrhage (sICH) was defined as any type of ICH with clinical worsening of 4 or more points on the NIHSS.

Statistical analyses were conducted using the Kruskal–Wallis ANOVA for continuous variables and a Chi-square test for categorical variables. For comparisons with NFZ data, the Wilcoxon test was employed.

## 3. Results

The analysis encompassed a total population of 250 patients. A statistical analysis and comparison were conducted between the first 100 patients and the subsequent 150 patients. For this analysis, the entire cohort was further stratified into two distinct schemes: patients admitted directly to a TCSC and those admitted via the drip-and-ship model from PSC. Another comparison focused on patients treated within an early 6 h time window versus those treated within an extended time window (6–24 h), based on the criteria outlined above. All results are presented in tables.

In a comparative analysis of the initial 100 and subsequent 150 individuals (see Table 2) within the baseline population, a significant disparity was observed in the prevalence of tobacco addiction (25% vs. 43.6%, *p* < 0.05) and the site of occlusion, with a higher incidence of internal carotid artery (ICA) occlusions in the first group (37% vs. 24.6%, *p* < 0.05). No significant differences were identified in terms of age, gender, arterial hypertension, atrial fibrillation, diabetes prevalence, or initial neurological state (NIHSS). More patients in the first group were admitted via the direct admission scheme than via the drip-and-ship model (97% vs. 70%, *p* < 0.0001), and significantly fewer patients were treated within an extended time window in this group (8% vs. 17.3%, *p* < 0.05). Consequently, fewer patients in the second group qualified for intravenous thrombolysis (81% vs. 65.3%, *p* < 0.01). No significant differences were found in time intervals for onset-to-reperfusion, onset-to-LVO identification, door-to-groin, and door-to-recanalization, clearly indicating opportunities for further logistical improvements. A significant change was noted in the groin-to-first pass time (27 vs. 23 min, *p* < 0.05), indicating an increase in operator experience. Although the median and interquartile range of the groin-to-recanalization time showed a slight improvement in the second group, this change was not statistically significant. The door-to-CT time increased significantly in the second group (9 vs. 10.5 min, *p* < 0.05), which was attributed to the admission of more patients from outside stroke centers offering varying standards of stroke care. Procedurally, the only difference between the groups was the number of balloon guide catheters used (64% vs. 32.7%, *p* < 0.0001), with no differences in the number of passes or first-pass effects. Significant differences were not observed in the rate of peri- and post-procedural complications, which may indicate that the introduction of a newly trained operator did not adversely affect outcomes. No statistically significant differences were found in clinical and radiological outcomes between the groups, particularly for the NIHSS fast (24 h) improvement, 3-month follow-up mRS score, and mortality rate.

In a comparative analysis between patients admitted through the direct admission scheme (directly to our TCSC) and those following the drip-and-ship model (patients admitted from PCSs), no significant differences were observed in general group characteristics at admission (see Table 3), the rate of early versus extended time window treatment, or the penetration of intravenous thrombolysis (IVT). In the drip-and-ship group, time intervals measured from the “door time” (defined as admission to the first stroke center) were extended: door-to-groin (110 vs. 159 min, *p* < 0.001); door-to-recanalization (158 vs. 200 min, *p* < 0.001); door-to-CT (9 vs. 16.5 min, *p* < 0.001); door-to-IVT (21 vs. 43 min, *p* < 0.001). Aforementioned time differences (time to CT and IVT) were associated with different hospital standards of stroke care at other institutions. Notably, certain time intervals were significantly shorter in the drip-and-ship group: groin-to-first pass, 26 vs. 21 min (*p* < 0.05), and groin-to-recanalization, 42.5 vs. 33 min (*p* < 0.01). We attribute these shorter time intervals to the greater representation of drip-and-ship patients in the latter 150 patient group, correlating with the increased experience of the operators and the angio-suite team. There were no significant differences in procedural aspects, complication rates, final clinical outcomes, or mortality between the direct-admission and drip-and-ship groups.

No significant differences were observed in group characteristics between patients in the early (up to 6 h) time window and those treated in the extended time window, as defined by the criteria outlined above (see Table 4). However, significant differences were observed in the site of occlusion, with a higher incidence of M1 occlusion of the middle cerebral artery (MCA) and a lower incidence of internal carotid artery (ICA) occlusion, as well as other locations and tandem lesions (without significance). These differences are attributed to the extended time window criteria and a brain ischemia pathology favoring M1. Additionally, there was a notably higher risk of bleeding associated with aggressive antiplatelet therapy in cases of carotid artery stenting, which significantly influenced treatment decisions. In the extended-time-window group, the rate of intravenous thrombolysis (IVT) was lower (78.2% vs. 29.4%, *p* < 0.0001), and the time intervals for door-to-groin (117.5 vs. 150 min, *p* < 0.005), door-to-CT (10 vs. 19.5 min, *p* < 0.01), and door-to-IVT (25 vs. 77.5 min, *p* < 0.001) were prolonged. Conversely, the groin-to-first pass time was shorter (26.5 vs. 21 min, *p* < 0.005), which was associated with more patients with an extended time window being treated later (as presented in the second group of 150 patients) and increases to overall operator and team experience. In terms of procedural aspects, complication rates, and clinical and radiological outcomes, no significant differences were found between the two groups. Good functional recovery measured by a modified Rankin Scale (mRS) 0–2 was slightly lower in the extended-time-window group, but this difference was not statistically significant.

According to the NFZ, 55.2% of patients in the Pomeranian region were treated using the drip-and-ship model. In our cohort, comprising the second group of 150 patients, this model was employed in 30% of cases, while 70% were treated using the direct-admission model. This proportion has increased tenfold since the inception of our center, rising from 3% among the initial cohort of 100 patients. When comparing our parameters for the first cohort of 100 patients and the subsequent 150 patients with data from both the Pomeranian region and Poland, we observed reduced door-to-CT, door-to-IVT, and door-to-groin times. The baseline neurological status of patients, as measured by the NIHSS, was more severe in both our cohorts compared to that among the general Pomeranian and Polish populations. However, changes in the NIHSS scores at patient discharge were more pronounced in our study subgroups (see Table 5).

## 4. Discussion

The primary objective of this study was to elucidate any differences between the initial cohort of 100 patients treated at the newly established TCSC and a subsequent cohort of 150 patients treated during the period when a second, newly qualified operator was involved, during which time the center also commenced 24/7 service, thereby becoming a high-volume center (defined as >100 patients per year). The initial experiences of our TCSC and the outcomes of the first procedures were compared to the HERMES registry and published in a separate article [4].

In all analyzed subgroups, there were no significant differences in pre-morbid characteristics, except for a notably higher prevalence of tobacco addiction in the second subgroup of 150 patients. As previously explained, more patients were initially treated through direct admission due to the establishment of the TCSC and reimbursement issues. These factors, along with the publication of new extended time window guidelines, resulted in fewer patients being treated through a direct model and a higher percentage of patients receiving IVT in the first group.

The improved skills of the neurointerventionalists were evident when groin-to-first pass times were compared between the two subgroups. Procedural times were found to be significantly shorter over time. As a positive outcome, no deterioration was observed in radiological outcomes or clinical results between the analyzed groups, especially when our results (newly established TCSC) were compared with nationwide data. Studies indicate that a higher annual operator volume (exceeding 40 procedures per year) positively influences successful reperfusion, although this factor does not correlate with improved clinical outcomes or affect complication rates [11]. When the results of only new operators were assessed, employing a stent retriever resulted in a higher recanalization rate, and the procedure times were shorter compared to those in the ADAPT group. This effect was evident in both team experience and individual operator experience [12]. A higher recanalization rate with a stent retriever was achievable with less clinical experience, but procedure times were significantly shorter with increased experience in the stent retriever-treated group. Additionally, the percentage of patients requiring additional therapy after an unsuccessful pass was higher in the ADAPT group than in the stent retriever group [11]. Our default technique, SAVE, is a maximalist approach that combines the benefits of suction with a distal access catheter and a stent retriever, with retraction of the system in one item.

In most publications, the analysis of operator experience was based on the successful recanalization rate (defined as TICI 2b or 3), procedure duration time (in minutes), and frequency of serious adverse events [13]. A higher level of experience is correlated with improved recanalization rates and a reduced procedural duration in MT operations [13]. In the cohort analyzed, both the TICI score and incidence of adverse events were at an acceptable level.

We trained an interventional neurologist involved in 24/7 rotations as an independent operator. Notably, only 14% of low-income countries report the availability of neurointerventional training, in contrast to 80% of high-income countries [14]. Training vascular neurologists in endovascular therapy (EVT) enhances access to this essential intervention, ensures comprehensive stroke care, promotes better access to optimal stroke care, and reduces service fragmentation [14]. The training of neurointerventionalists is guided by established protocols, such as those from the World Federation for Interventional Stroke Treatment (WIST) [15]. As demonstrated by the results, collaboration between interventional cardiologists and neurologists is an effective strategy for expanding thrombectomy-capable stroke centers (TCSCs) in Poland.

In a comparative analysis of patients admitted directly versus those admitted via the drip-and-ship model, we observed extended time intervals associated with both patient transfers and intrahospital processes such as door-to-CT and door-to-IVT times. These findings underscore the urgent need to improve both intra- and extra-hospital patient workflows. Globally, stroke intervention centers have implemented various strategies to optimize their EVT workflows, resulting in significant time reductions and potentially improved outcomes for patients with AIS [16]. Additionally, in transfer-mode patients, enabling a single stroke physician to decide whether to operate, rather than requiring both an interventionist and a neurologist, decreased door-to-groin time by 47%. Additionally, conducting after-transfer neurological assessments at the angio-suite, rather than the emergency department, reduced time by 32% [16].

Previous analyses indicate that each 15 min reduction in door-to-puncture time correlates with an increased likelihood of improved quality of life and independence, as assessed by the EQ-5D-5L [17] and MRS. A door-to-puncture time of less than 60 min (the result still not obtained in our population) is associated with improved outcomes in domains such as pain, discomfort, and mobility [17]. Additional studies suggest that a shorter door-to-puncture EVT time corresponds to better outcomes in health-related quality of life across all domains. These findings underscore the positive impact of expedited door-to-treatment times on patient-reported outcomes and underscore the need for initiatives to enhance patient-centered care in acute stroke by optimizing in-hospital workflows [17].

The advantages of high-quality stroke care in mitigating mortality risk are substantially diminished by delays in hospital admission, examination, and thrombolysis [17]. The timeliness and quality of AIS care are affected by geographic location, admission NIHSS scores, and comorbidity profiles [18].

Although the time window can be expanded up to 24 h for certain acute stroke patients, time remains a critical determinant of outcomes [19]. Several factors will affect timely access for the majority of MT candidates, including prehospital management systems, transportation models, in-hospital workflow organization, the accreditation and infrastructure of centers, the training of neurointervention professionals, and the geographic distribution of centers [19].

Notably, in the drip-and-ship subgroup, the significant reduction in time from groin puncture to first pass and recanalization indirectly reflects an improvement in the operators’ level of experience, as this subgroup was predominantly treated in the latter 150 cases.

The prenotification process is a critical component in the comprehensive management of stroke patients within hospital settings and is associated with a higher likelihood of reduced time to treatment for both intravenous thrombolysis and endovascular therapy (EVT) [20]. This practice is currently standard at our TCSC and at the Primary Stroke Centers (PSCs) with which we collaborate.

The observed increase in patient volume due to new cooperation with the surrounding PSCs and the implementation of a drip-and-ship model did not affect recanalization rates (TICI 2b-3), as demonstrated in other studies [5]. Procedural volume, moreover, does not predict long-term functional outcomes in established and regularly utilized EVT facilities. High-volume centers mitigate lower primary admission rates and transfer delays through increased efficiency and expedited in-house procedural times. However, the overall time from symptom onset to flow restoration did not significantly differ between high- and low-volume centers, except in subgroup analyses that adjusted for primary admission to the MT center [5]. Other single-center studies found no significant differences in functional independence rates between the drip-and-ship and mothership (or direct admission) models [21]. Although the indirect model was associated with an increased risk of hemorrhagic transformation and symptomatic intracranial hemorrhage, the findings did not support a strategy that involves bypassing the nearest primary stroke centers when considering overall outcomes [21].

A recent meta-analysis indicated that the mothership paradigm might be superior to the drip-and-ship approach in terms of achieving functional independence, with rates of 53% compared to 47%, respectively. However, similar rates of recanalization, symptomatic intracerebral hemorrhage (sICH), and mortality at 90 days have been documented under both approaches [22].

The duration time from symptom onset to groin puncture and subsequent reperfusion was significantly extended among the transfer group in another study [23]. However, the times from groin puncture to reperfusion and from door to reperfusion were comparable between the two models [23]. The rates of successful reperfusion, symptomatic intracranial hemorrhage (sICH), discharge to home, and in-hospital mortality were also similar. Thus, despite the observed significantly prolonged intervals from symptom onset to the initiation of mechanical thrombectomy in transferred patients in our study, these factors did not affect the prognosis [23].

The publication of clinical trial results related to extended time windows and the treatment of large core strokes has led to changes in guidelines and, consequently, a significant increase in the number of patients receiving endovascular treatment. In parallel, there has been a substantial rise in the number of patients with poorer general health and more comorbidities qualified for MT. An analysis of the German Registry of Stroke Patients (2017–2021) indicated significant changes in treatment approaches. Patients qualified for mechanical thrombectomy were older and less severely affected than those in the past [24]. There was a notable increase in both the number of patients treated more than six hours after they were last seen healthy and patients with only medium-sized vessel occlusions (MEVO). Notably, as the use of intravenous thrombolysis decreased, the rate of good functional outcomes (mRS 0–2) also declined. Additionally, mortality at three months increased despite improvements in technical (radiological) outcomes [24]. This result indirectly demonstrates that we still require more sophisticated tools to identify patients who will benefit from MT. Ultimately, the indications for EVT have expanded, but the higher mortality rates observed may reflect a willingness to treat patients with more severe general health conditions [24]. In our late-time-window subgroups, we also observed lower intravenous thrombolysis penetration. Additionally, prolonged time intervals (door-to-groin and door-to-IVT) due to more complex neuroimaging procedures (MRI, CTP) were noted, but neither adversely affected clinical outcomes nor increased the rate of complications. In our observations, the shorter mean groin-to-first pass time was attributed to an increase in operator experience and a higher incidence of M1 middle cerebral artery occlusions in the subgroup of patients treated later. The proximal thrombus location was considered easier to reach than, for example, internal carotid artery or M2 occlusions.

The comparison between the clinical and radiological results observed in our cohort and general regional (Pomerania) and Polish data, as published by the NFZ, underscores the significant role of TCSC within the overall system of endovascular treatment for AIS patients in Poland. Moreover, mechanical thrombectomy is cost-effective in improving patient outcomes, reducing length of hospitalization and significantly reducing the cost of thrombectomy, providing hospital [25].

The limitation of the presented study is its retrospective, single-center design, limited sample in some analyzed subgroups and univariate statistical analyses.

## 5. Conclusions

In this study, we presented technical and clinical data regarding the interventional treatment of acute ischemic stroke patients with large vessel occlusion at a newly established thrombectomy-capable stroke center (TCSC) in Poland. This study focused on ongoing changes in qualification guidelines, the transition from a ‘direct-admission-only’ model to a mixed population involving treatment under both the ‘direct’ and ‘drip-and-ship’ models, and the learning curve of the interventional stroke team. The comparison between the first 100 and subsequent 150 MT-treated patients highlighted significant differences over time. These differences included changes in the admission criteria, time of acceptance to treatment, and intravenous thrombolysis qualification. Time intervals and procedural aspects presented a learning curve effect with expected improvements in the second group, indicating an enhanced operator experience. Comparative analysis between the direct-admission and drip-and-ship models revealed extended time intervals in the latter. Patients in the extended-time-window group had lower intravenous thrombolysis rates and prolonged door-to-groin, door-to-CT, and door-to-IVT times. This study highlights the need to optimize patient workflows and the potential positive impacts of TCSCs within the overall endovascular treatment system for acute ischemic stroke patients in Poland.

## Figures and Tables

**Table 1:** Extended time window criteria, based on Wake-up [6], DAWN [7] and DEFUSE 3 [8] trials.

	WAKE-UP	DAWN	DEFUSE 3
Inclusion criteria	LKW > 4.5 h Age 18–80 years Acute stroke MRI including DWI and FLAIR showing a pattern of DWI–FLAIR-mismatch, i.e., acute ischemic lesion visible on DWI but no marked parenchymal hyperintensity visible on FLAIR, indicative of acute ischemic lesions ≤ 4.5 h	<1/3 MCA territory involved, as evidenced by CT or MRI Occlusion of the intracranial ICA and/or MCA-M1, as evidenced by MRA or CTA Clinical Imaging Mismatch (CIM) defined as one of the following on RAPID MR-DWI or CTP-rCBF maps: 0–20 cc core infarct and NIHSS ≥ 10 (and age ≥ 80 years old) 0–30 cc core infarct and NIHSS ≥ 10 (and age < 80 years old) 31 cc to < 50 cc core infarct and NIHSS ≥ 20 (and age < 80 years old)	ICA or MCA-M1 occlusion (carotid occlusions can be cervical or intracranial, with or without tandem MCA lesions) on MRA or CTA and Target Mismatch Profile on CT perfusion or MRI (ischemic core volume < 70 mL, mismatch ratio > 1.8, and mismatch volume > 15 mL)

**Table 2:** Comparison between groups of the first 100 and subsequent 150 patients.

	First 100	Second 150	*p*
Age [y]; median (IQR)/N	70.5 (62–78)/100	71 (65–78)/150	0.7505
Sex [female]; n/N (%)	43/100 (43%)	72/150 (48%)	0.4380
**Clinical characteristic at admission**			
Pre mRS; median (IQR)/N	0 (0–0)/100	0 (0–1)/100	0.1189
Pre mRS ≤ 2; n/N (%)	99/100 (99%)	148/150 (98.7%)	0.8129
Arterial hypertension; n/N (%)	85/100 (85%)	133/150 (89.3%)	0.3190
Diabetes; n/N (%)	24/100 (24%)	49/149 (32.9%)	0.1318
***Smoking; n/N (%)***	***25/100 (25%)***	***65/149 (43.6%)***	***0.0028***
Atrial fibrillation; n/N (%)	48/100 (48%)	72/149 (49%)	0.8781
NIHSS baseline; median (IQR)/N	17 (11–23)/100	16 (11–21)/150	0.2830
**Occlusion site**			
M1; n/N (%)	40/100 (40%)	52/150 (34.7%)	0.3926
M2; n/N (%)	8/100 (8%)	21/150 (14%)	0.1475
***ICA; n/N (%)***	***37/100 (37%)***	***37/150 (24.7%)***	***0.0367***
Tandem anterior circ.; n/N (%)	12/100 (12%)	29/150 (19.3%)	0.1258
BA/VA/PCA; n/N (%)	3/100 (3%)	11/150 (7.3%)	0.1451
Occlusion side; n/N (%)	Left 57/100 (57%) Right 40/100 (40%) Posterior 3/100 (3%)	Left 85/150 (56.7%) Right 55/150 (36.7%) Posterior 10/150 (6.7%)	0.4215
**Admission scheme**			
***Direct*** **vs. *Drip-n-Ship; n/N (%)***	***97/100 (97%)***	***105/150 (70%)***	***<0.0001***
***Wake-up strokes; n/N (%)***	***8/100 (8%)***	***26/150 (17.3%)***	***0.0353***
***Intravenous thrombolysis; n/N (%)***	***81/100 (81%)***	***98/150 (65.3%)***	***0.0072***
Hospitalization (days); median (IQR)/N	9 (8–11)/100	9 (8–14)/149	0.5370
**Time intervals**			
Onset-to-reperfusion [min]; med.(IQR)/N	265 (215.5–319.75)/91	249 (202–312)/126	0.4334
Onset-to-LVO [min]; median (IQR)/N	89 (65–133.75)/91	95 (65.25–147.75)/127	0.4879
Door-to-groin [min]; median (IQR)/N	119 (97–150)/98	120.5 (83.5–164.5)/144	0.6433
Door-to-recanalization [min]; med (IQR)/N	169 (142.25–218.25)/99	167 (125.75–214.25)/145	0.5845
***Groin-to-first pass [min]; med (IQR)/N***	***27 (22–43.5)/100***	***23 (17–36)/150***	***0.0196***
Groin-to-recanalization [min]; med(IQR)/N	42.5 (32.5–79)/100	39.5 (25–63)/150	0.1345
***Door-to-CT [min]; median (IQR)/N***	***9 (5–15.75)/99***	***10.5 (5–23)/150***	***0.0401***
Door-to-IVT [min]; median (IQR)/N	23 (15–36)/81	25.5 (15–46)/98	0.1978
LVO-to-groin [min]; median (IQR)/N	107 (88.25–127.5)/99	96.5 (72–133)/150	0.1544
**Type of anesthesia**			
GA; n/N (%)	57/100 (57%)	79/150 (51.7%)	0.5012
**Procedural aspects**			
Number of passes; median (IQR)/N	1 (1–2.5)/100	1 (1–2)/149	0.3623
First pass effect; n/N (%)	44/99 (44.4%)	71/150 (47.3%)	0.6552
***BGC used; n/N (%)***	***64/100 (64%)***	***49/150 (32.7%)***	***<0.0001***
GP IIb/IIIa; n/N (%)	11/100 (11%)	13/150 (8.7%)	0.5403
**Peri- and post-procedural complications**			
ICH; n/N (%)	18/100 (18%)	31/150 (20.7%)	0.6036
PH2; n/N (%)	8/100 (8%)	17/150 (11.3%)	0.3904
sICH; n/N (%)	10/100 (10%)	18/150 (12%)	0.6240
Periprocedural hemorrhage; n/N (%)	2/100 (2%)	5/150 (3.3%)	0.5321
**Clinical and radiological outcomes**			
TICI 2b-3; n/N (%)	94/100 (94%)	138/150 (92%)	0.5498
TICI 3; n/N (%)	51/100 (51%)	76/150 (50.7%)	0.9589
mRS 0–1; n/N (%)	32/100 (32%)	38/150 (25.3%)	0.2510
mRS 0–2; n/N (%)	54/100 (54%)	64/150 (42.7%)	0.0793
Death; n/N (%)	18/100 (18%)	23/150 (15.3%)	0.5777
NIHSS fast improvement (24 h); median (IQR)/N	8.5 (4–17.5)/96	12 (5–19.75)/147	0.083779
NIHSS final; median (IQR)/N	4 (2–10)/83	5 (2–11)/126	0.3253

**Table 3:** Comparison between patients treated under direct TCSC admission and the drip-and-ship model.

	Direct Admission	Drip-and-Ship	*p*
Age [y]; median (IQR)/N	70 (62–78)/202	73 (65–77.5)/48	0.4614
Sex [female]; n/N (%)	92/202 (45.4%)	23/48 (47.9%)	0.7674
**Clinical characteristic at admission**			
Pre mRS; median (IQR)/N	0 (0–0)/202	0 (0–1)/48	0.3877
Pre mRS ≤ 2; n/N (%)	198/200 (99%)	47/48 (97.9%)	0.5326
Arterial hypertension; n/N (%)	175/201 (80.3%)	43/48 (89.6%)	0.6356
Diabetes; n/N (%)	59/201 (29.4%)	14/48 (29.2%)	0.9797
Smoking; n/N (%)	71/201 (35.3%)	19/48 (39.6%)	0.5817
Atrial fibrillation; n/N (%)	103/201 (51.2%)	18/48 (37.5%)	0.0876
NIHSS baseline; median (IQR)/N	17 (12–22)/202	17 (10–20.5)/48	0.3907
**Occlusion site**			
M1; n/N (%)	71/202 (35.1%)	21/48 (43.7%)	0.2676
M2; n/N (%)	25/202 (12.4%)	4/48 (8.3%)	0.4326
ICA; n/N (%)	61/202 (30.2%)	13/48 (27.1%)	0.6715
Tandem anterior circ.; n/N (%)	34/202 (16.8%)	7/48 (14.6%)	0.7059
BA/VA/PCA; n/N (%)	11/202 (5.4%)	3/48 (6.2%)	0.8279
Left side in anterior circ.; n/N (%)	Left 117/202 (57.9%) Right 75/202 (37.1%) Posterior 10/202 (5%)	Left 25/48/(52.1%) Right 20/48 (41.7%) Posterior 3/48 (6.2%)	0.7525
**Admission scheme**			
Wake up strokes; n/N (%)	24/202 (11.9%)	10/48 (20.8%)	0.1046
Intravenous thrombolysis; n/N (%)	148/202 (73.3%)	31/48 (64.6%)	0.2313
Hospitalization (days); median (IQR)/N	9 (8–13)/201	9 (8–11)/48	0.6524
**Time intervals**			
Onset-to-reperfusion; med. (IQR)/N	252 (203–309.75)/179	272.5 (224–380)/38	0.0958
Onset-to-LVO [min]; med. (IQR)/N	94 (66–142.5)/180	75.5 (55–172)/38	0.2659
***Door-to-groin [min]; med. (IQR)/N***	***110 (85–138)/195***	***159 (133.5–205.75)/47***	***<0.000001***
***Door-to-recanalization;*** ***med.*** ***(IQR)/N***	***158 (125.75–207)/197***	***200 (168–247)/47***	***0.000018***
***Groin-to-first pass [min]; med.*** ***(IQR)/N***	***26 (20–44)/202***	***21 (16–31.5)/48***	***0.012284***
***Groin-to-recanalization;*** ***med. (IQR)/N***	***42.5 (30–77)/202***	***33 (25–51)/48***	***0.008742***
***Door-to-CT [min]; med. (IQR)/N***	***9 (5–17)/201***	***16.5 (10–26.5)/48***	***0.000064***
***Door-to-IVT [min]; med. (IQR)/N***	***21 (15–35)/148***	***43 (32.5–66.25)/31***	***0.000002***
***LVO-to-groin [min]; med. (IQR)/N***	***94 (72.75–117.25)/201***	***146 (118.5–184.5)/48***	***<0.000001***
**Type of anesthesia**			
GA; n/N (%)	113/202 (55.9%)	23/48 (47.9%)	0.3167
**Procedural aspects**			
Number of passes; med. (IQR)/N	1 (1–2.25)/201	1 (1–2)/48	0.9760
First pass effect; n/N (%)	94/201 (46.8%)	21/48 (43.7%)	0.7070
BGC used; n/N (%)	97/202 (48%)	16/48 (33.3%)	0.0666
**Peri- and post-procedural complication**			
ICH; n/N (%)	41/202 (20.3%)	8/48 (19.2%)	0.5698
PH2; n/N (%)	19/202 (9.4%)	6/48 (12.5%)	0.5215
sICH; n/N (%)	23/202 (11.4%)	5/48 (10.4%)	0.8485
Periprocedural hemorrhage; n/N (%)	6/202 (3%)	1/48 (2.1%)	0.7383
**Clinical outcome**			
TICI 2b-3; n/N (%)	188/202 (93.1%)	44/48 (91.7%)	0.7359
TICI 3; n/N (%)	21/123 (17.1%)	27/127 (21.3%)	0.4017
mRS 0–1; n/N (%)	55/202 (27.2%)	15/48 (31.2%)	0.5777
mRS 0–2; n/N (%)	86/132 (65.2%)	64/118 (54.2%)	0.0793
Death; n/N (%)	34/202 (16.8%)	7/48 (14.6%)	0.7059
NIHSS fast improvement (24 h); med. (IQR)/N	10 (4.25–19)/195	11 (5–17)/48	0.891367
NIHSS final; med. (IQR)/N	5 (2–10.75)/167	4 (2–10)/42	0.731223

**Table 4:** Comparison between the early and extended time windows.

	Early Time Window	Extended Time Window	*p*
Age, y (SD)	70 (63–78)/216	72 (66–76)/34	0.7769
Sex, female, n (%)	19/34 (55.9%)	96/216 (44.4%)	0.2145
**Clinical characteristic at admission**			
Pre mRS; median (IQR)/N	0 (0–0)/215	0 (0–0)/34	0.5272
Pre mRS ≤ 2; n/N (%)	213/215 (99.1%)	33/34 (97.1%)	0.3189
Arterial hypertension; n/N (%)	186/214 (86.3%)	31/34 (91.2%)	0.4862
Diabetes; n/N (%)	62/215 (28.8%)	11/34 (32.4%)	0.6762
Smoking; n/N (%)	78/215 (36.3%)	12/34 (35.3%)	0.9117
Atrial fibrillation; n/N (%)	104/215 (48.4%)	17/34 (50%)	0.8602
NIHSS baseline; median (IQR)/N	17 (12–22)/216	15 (10–19)/34	0.1740
**Occlusion site**			
***M1; n/N (%)***	***73/216 (33.8%)***	***19/34 (55.9%)***	***0.0132***
M2; n/N (%)	27/216 (12.5%)	2/34 (5.9%)	0.2636
***ICA; n/N (%)***	***69/216 (31.9%)***	***5/34 (14.7%)***	***0.0411***
Tandem anterior circ.; n/N (%)	37/216 (17.1%)	4/34 (11.8%)	0.4332
BA/VA/PCA; n/N (%)	10/216 (4.6%)	4/34 (11.8%)	0.0932
Left side in anterior circ.; n/N (%)	Left 121/216 (56%) Right 86/216 (39.8%) Posterior 9/216/(4.2%)	Left 21/34/(61.8%) Right 9/34 (26.5%) Posterior 4/34 (11.8%)	0.0903
**Admission scheme**			
Direct (vs. Drip-n-Ship); n/N (%)	178/216 (82.4%)	24/34 (70.6%)	0.1046
***Intravenous thrombolysis; n/N (%)***	***169/216 (78.2%)***	***10/34 (29.4%)***	***<0.0001***
Hospitalization (days); median (IQR)/N	9 (8–13)/215	9 (8–12)/34	0.7636
**Time intervals**			
Onset-to-reperfusion [min]; median (IQR)/N	253.5 (204–318)/214	282 (201–381)/3	0.7423
Onset-to-LVO [min]; median (IQR)/N	91.5 (65–143)/214	82 (65–152.5)/4	0.8729
***Door-to-groin [min]; median (IQR)/N***	***117.5 (86.5–148)/208***	***150 (101–195)/34***	***0.001876***
Door-to-recanalization [min]; median (IQR)/N	164 (130–211)/210	193 (157–228)/34	0.074025
***Groin-to-first pass [min]; median (IQR)/N***	***26.5 (20–43.5)/216***	***21 (15–26)/34***	***0.003839***
Groin-to-recanalization [min]; median (IQR)/N	40.5 (30–67)/216	32 (23–72)/34	0.138818
***Door-to-CT [min]; median (IQR)/N***	***10 (5–17.75)/215***	***19.5 (6–26)/34***	***0.005656***
***Door-to-IVT [min]; median (IQR)/N***	***25 (15–40)/160***	***77.5 (62–94)/10***	***0.000208***
***LVO-to-groin [min]; median (IQR)/N***	***99 (77–127)/215***	***121 (92–170)/34***	***0.013740***
**Type of anesthesia**			
GA; n/N (%)	118/216 (54.6%)	18/34 (52.9%)	0.8545
**Procedural aspects**			
Number of passes; median (IQR)/N	1 (1–2)/215	1.5 (1–2)/34	0.7415
First pass effect; n/N (%)	99/215 (46%)	16/34 (47.1%)	0.9126
BGC used; n/N (%)	100/216 (46.3%)	13/34 (38.2%)	0.3810
**Peri- and post-procedural complication**			
ICH; n/N (%)	41/216 (19%)	8/34 (23.5%)	0.5355
PH2; n/N (%)	22/216 (10.2%)	3/34 (8.8%)	0.8061
sICH; n/N (%)	25/216 (11.6%)	3/34 (8.8%)	0.6371
Periprocedural hemorrhage; n/N (%)	7/216 (3.2%)	0/34 (0%)	0.2880
**Angiographic and clinical outcome**			
TICI 2b-3; n/N (%)	199/216 (92.1%)	33/34 (97.1%)	0.3023
TICI 3; n/N (%)	112/216 (51.9%)	15/34 (44.1%)	0.4027
mRS 0–1; n/N (%)	63/216 (29.2%)	7/34 (20.6%)	0.3014
mRS 0–2; n/N (%)	105/216 (48.6%)	13/34 (38.2%)	0.2609
Death; n/N (%)	38/216 (17.6%)	3/34 (8.8%)	0.2002
NIHSS fast improvement (24 h); median (IQR)/N	10 (5–19)/209	11 (5–18)/34	0.745917
NIHSS final; median (IQR)/N	4 (2–10)/178	8 (3–12)/31	0.196175

**Table 5:** Comparison between the two subgroups of our cohort and NFZ data (source: https://ezdrowie.gov.pl/portal/home/badania-i-dane/zdrowe-dane/raporty/nfz-o-zdrowiu-udar-niedokrwienny-mozgu URL accessed on 4 February 2026) [10].

	First 100 (Mean; min, pts)		Second 150 (Mean; min, pts)	
**Door-to-CT**	11.6		17.0	
Pomeranian region	68.77	*p* < 0.001	68.77	*p* < 0.001
Poland	56.55	*p* < 0.001	56.55	*p* < 0.001
**Door-to-IVT**	24.8		25.8	
Pomeranian region	64.79	*p* < 0.001	64.79	*p* < 0.001
Poland	74.23	*p* < 0.001	74.23	*p* < 0.001
**Door-to-groin**	126		126	
Pomeranian region	165.69	*p* < 0.001	156.69	*p* < 0.001
Poland	152.93	*p* < 0.001	152.93	*p* < 0.001
**NIHSS baseline**	17		16.1	
Pomeranian region	14.69	*p* < 0.001	14.69	*p* < 0.05
Poland	14.80	*p* < 0.001	14.80	*p* < 0.05
**NIHSS change**	−10.1		−8.5	
Pomeranian region	−6.81	*p* < 0.001	−6.81	*p* < 0.001
Poland	−6.35	*p* < 0.001	−6.35	*p* < 0.001

## Data Availability

The data presented in this study are available on request from the corresponding author due to ethical and legal reasons.
